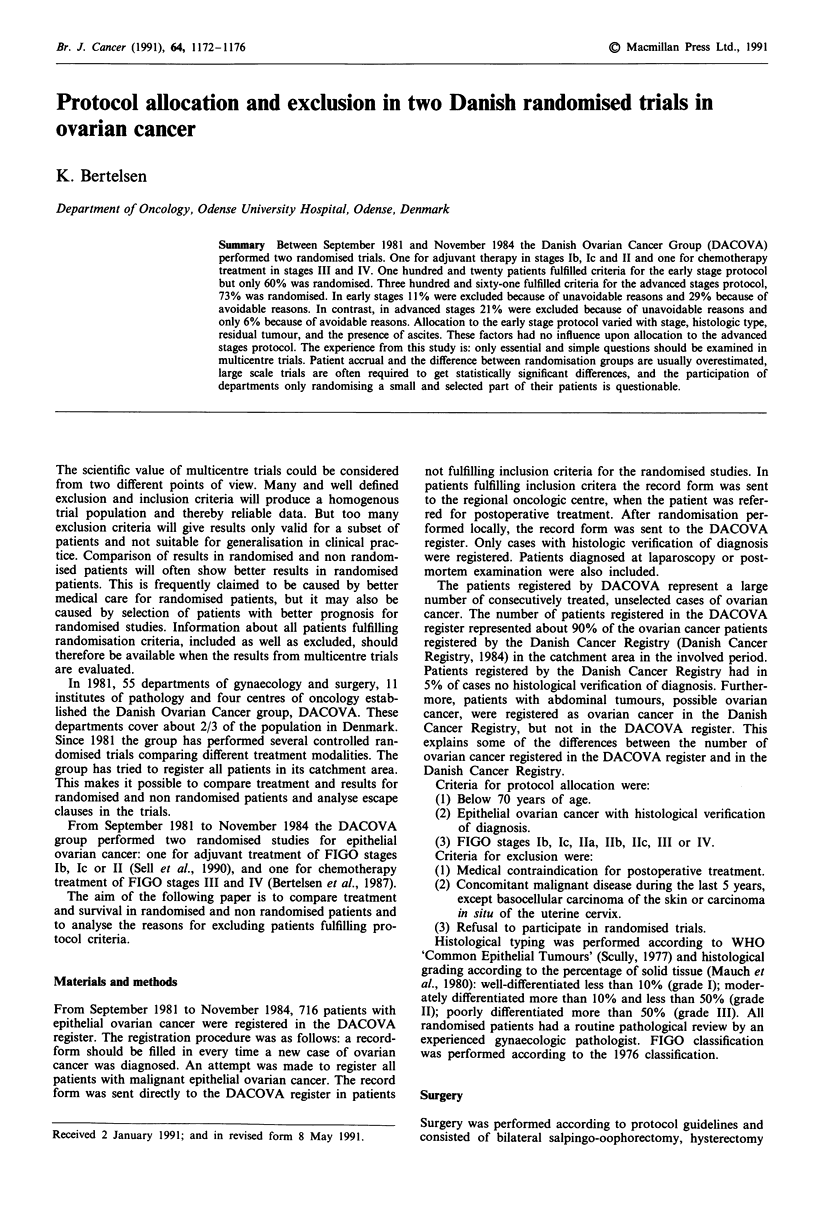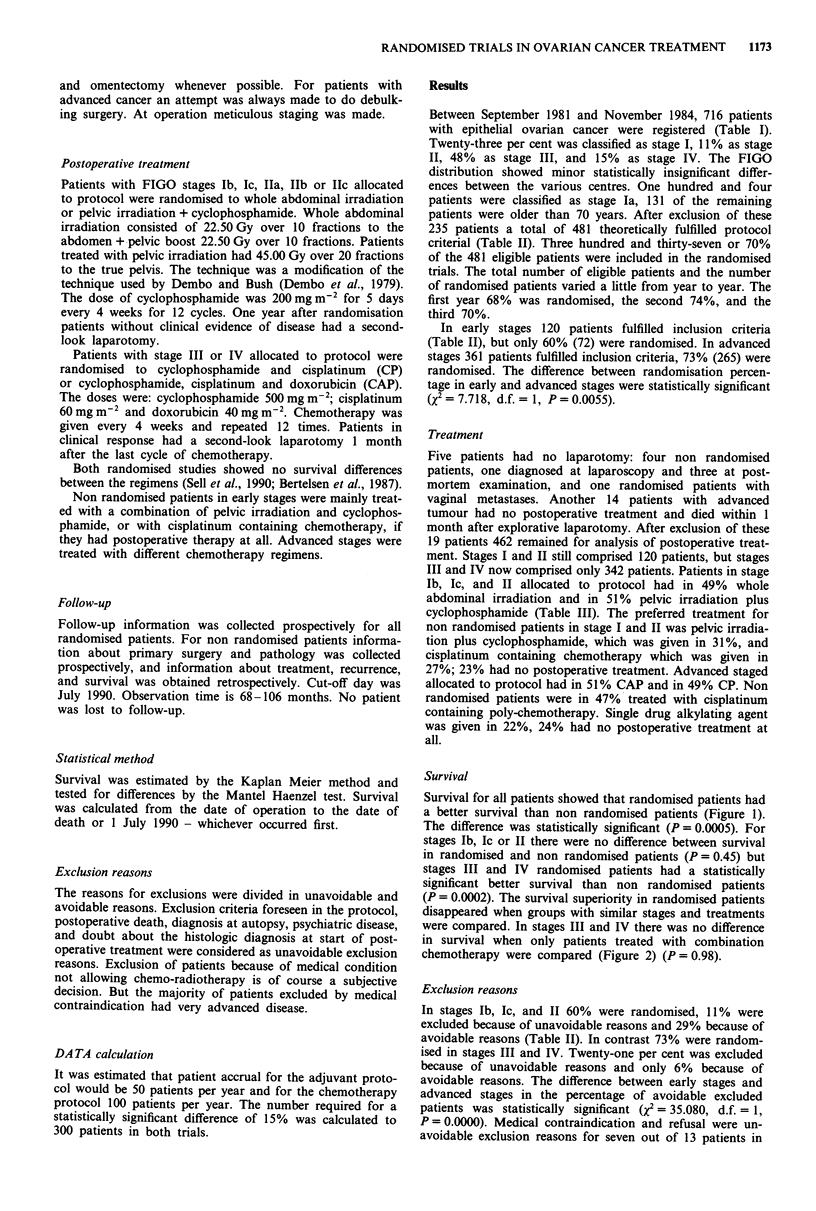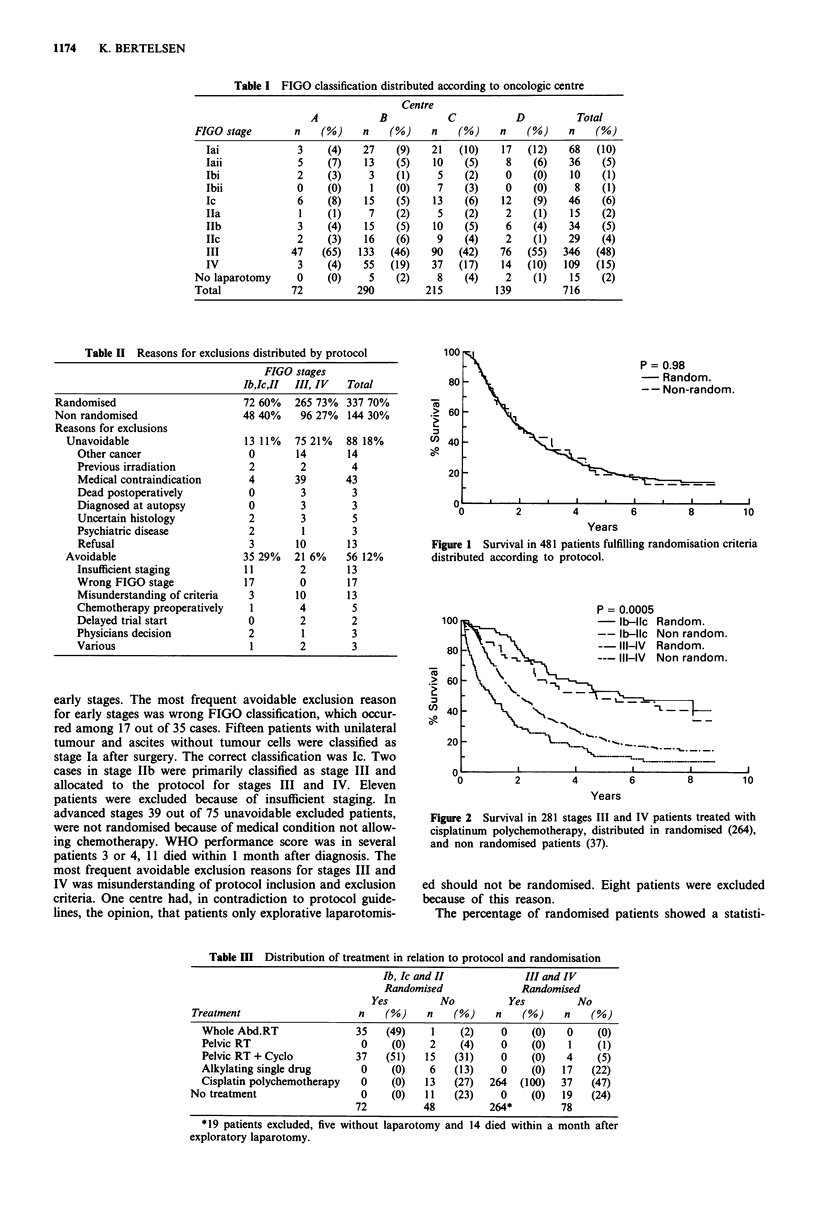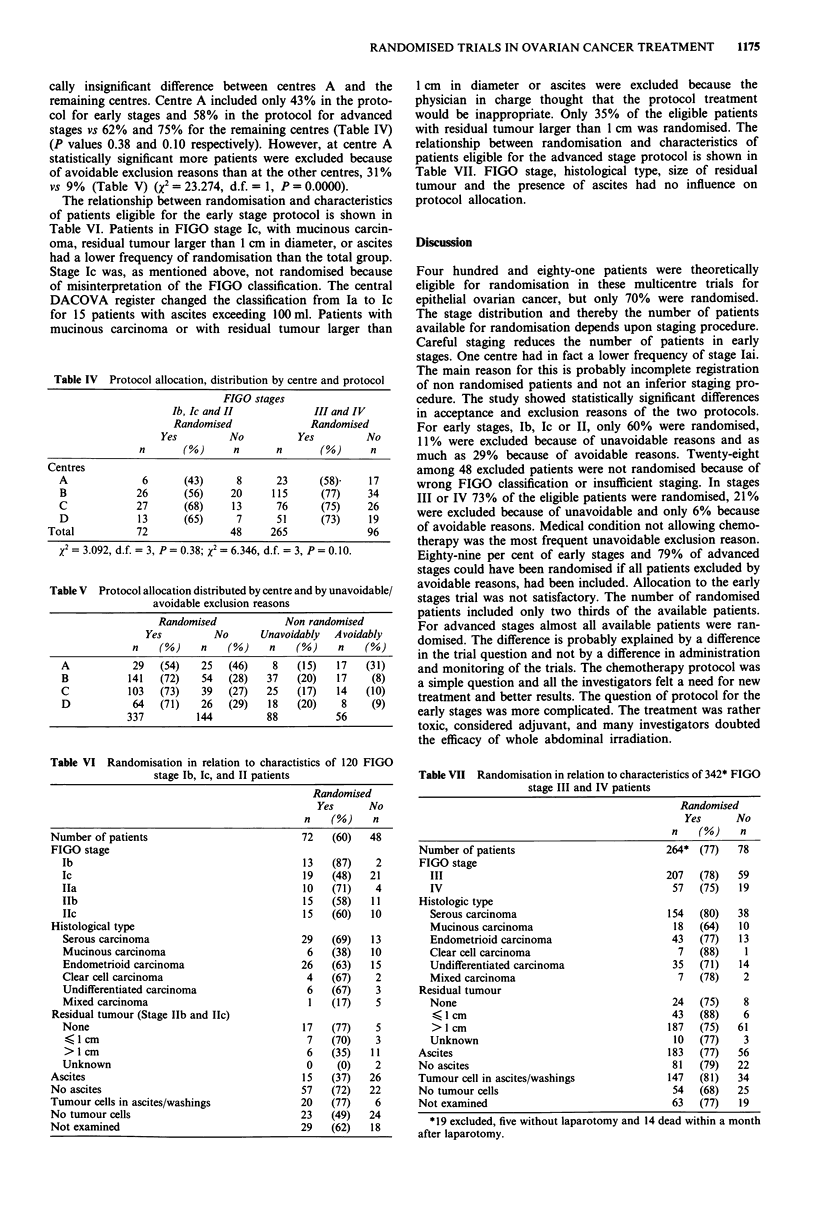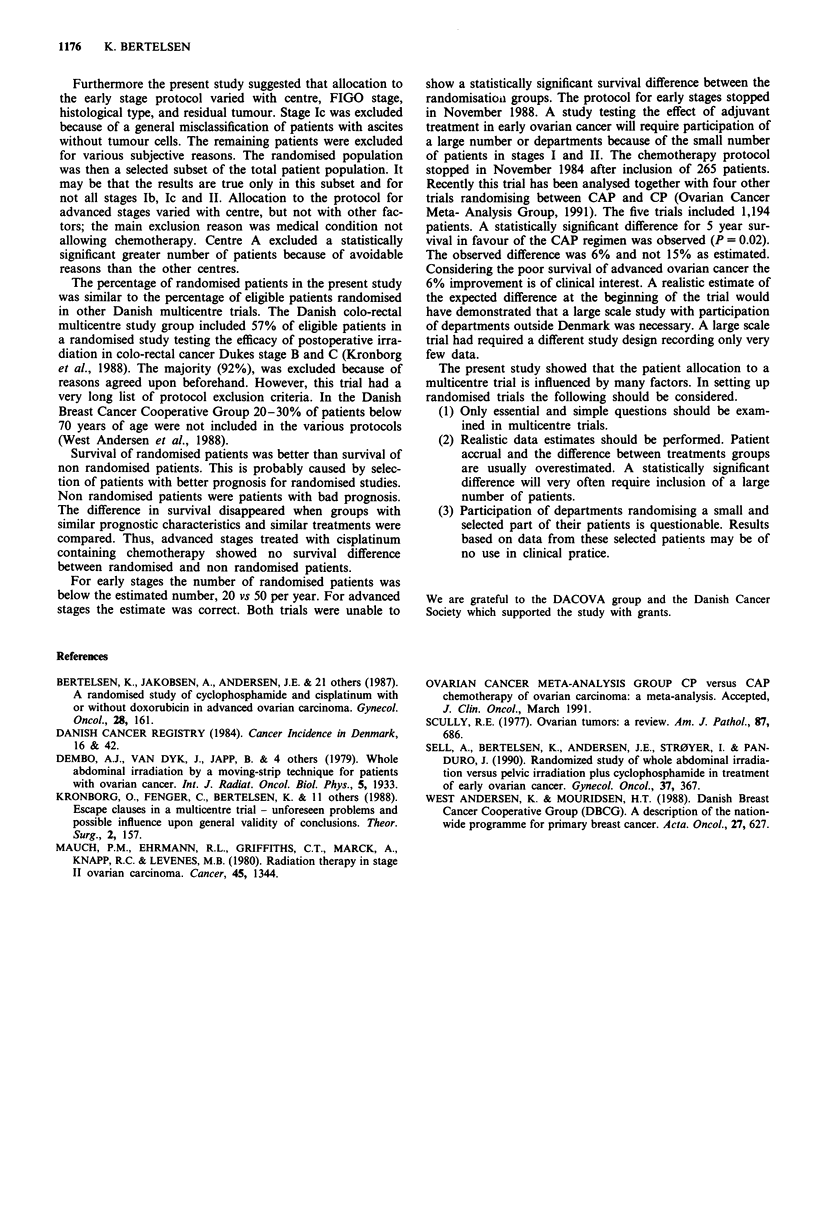# Protocol allocation and exclusion in two Danish randomised trials in ovarian cancer.

**DOI:** 10.1038/bjc.1991.485

**Published:** 1991-12

**Authors:** K. Bertelsen

**Affiliations:** Department of Oncology, Odense University Hospital, Denmark.

## Abstract

Between September 1981 and November 1984 the Danish Ovarian Cancer Group (DACOVA) performed two randomised trials. One for adjuvant therapy in stages Ib, Ic and II and one for chemotherapy treatment in stages III and IV. One hundred and twenty patients fulfilled criteria for the early stage protocol but only 60% was randomised. Three hundred and sixty-one fulfilled criteria for the advanced stages protocol, 73% was randomised. In early stages 11% were excluded because of unavoidable reasons and 29% because of avoidable reasons. In contrast, in advanced stages 21% were excluded because of unavoidable reasons and only 6% because of avoidable reasons. Allocation to the early stage protocol varied with stage, histologic type, residual tumour, and the presence of ascites. These factors had no influence upon allocation to the advanced stages protocol. The experience from this study is: only essential and simple questions should be examined in multicentre trials. Patient accrual and the difference between randomisation groups are usually overestimated, large scale trials are often required to get statistically significant differences, and the participation of departments only randomising a small and selected part of their patients is questionable.


Br. J. Cancer (1991), 64, 1172-1176 © Macmillan Press Ltd., 1991

### Protocol allocation and exclusion in two Danish randomised trials in ovarian cancer

K. Bertelsen

*Department of Oncology, Odense University Hospital, Odense, Denmark*

**Summary** Between September 1981 and November 1984 the Danish Ovarian Cancer Group (DACOVA) performed two randomised trials. One for adjuvant therapy in stages Ib, Ic and II and one for chemotherapy treatment in stages III and IV. One hundred and twenty patients fulfilled criteria for the early stage protocol but only 60% was randomised. Three hundred and sixty-one fulfilled criteria for the advanced stages protocol, 73% was randomised. In early stages 11% were excluded because of unavoidable reasons and 29% because of avoidable reasons. In contrast, in advanced stages 21% were excluded because of unavoidable reasons and only 6% because of avoidable reasons. Allocation to the early stage protocol varied with stage, histologic type, residual tumour, and the presence of ascites. These factors had no influence upon allocation to the advanced stages protocol. The experience from this study is: only essential and simple questions should be examined in multicentre trials. Patient accrual and the difference between randomisation groups are usually overestimated, large scale trials are often required to get statistically significant differences, and the participation of departments only randomising a small and selected part of their patients is questionable.

The scientific value of multicentre trials could be considered from two different points of view. Many and well defined exclusion and inclusion criteria will produce a homogenous trial population and thereby reliable data. But too many exclusion criteria will give results only valid for a subset of patients and not suitable for generalisation in clinical practice. Comparison of results in randomised and non randomised patients will often show better results in randomised patients. This is frequently claimed to be caused by better medical care for randomised patients, but it may also be caused by selection of patients with better prognosis for randomised studies. Information about all patients fulfilling randomisation criteria, included as well as excluded, should therefore be available when the results from multicentre trials are evaluated.

In 1981, 55 departments of gynaecology and surgery, 11 institutes of pathology and four centres of oncology established the Danish Ovarian Cancer group, DACOVA. These departments cover about 2/3 of the population in Denmark. Since 1981 the group has performed several controlled randomised trials comparing different treatment modalities. The group has tried to register all patients in its catchment area. This makes it possible to compare treatment and results for randomised and non randomised patients and analyse escape clauses in the trials.

From September 1981 to November 1984 the DACOVA group performed two randomised studies for epithelial ovarian cancer: one for adjuvant treatment of FIGO stages Ib, Ic or II (Sell *et al.*, 1990), and one for chemotherapy treatment of FIGO stages III and IV (Bertelsen *et al.*, 1987).

The aim of the following paper is to compare treatment and survival in randomised and non randomised patients and to analyse the reasons for excluding patients fulfilling protocol criteria.

### Materials and methods

From September 1981 to November 1984, 716 patients with epithelial ovarian cancer were registered in the DACOVA register. The registration procedure was as follows: a record-form should be filled in every time a new case of ovarian cancer was diagnosed. An attempt was made to register all patients with malignant epithelial ovarian cancer. The record form was sent directly to the DACOVA register in patients not fulfilling inclusion criteria for the randomised studies. In patients fulfilling inclusion critera the record form was sent to the regional oncologic centre, when the patient was referred for postoperative treatment. After randomisation performed locally, the record form was sent to the DACOVA register. Only cases with histologic verification of diagnosis were registered. Patients diagnosed at laparoscopy or post-mortem examination were also included.

The patients registered by DACOVA represent a large number of consecutively treated, unselected cases of ovarian cancer. The number of patients registered in the DACOVA register represented about 90% of the ovarian cancer patients registered by the Danish Cancer Registry (Danish Cancer Registry, 1984) in the catchment area in the involved period. Patients registered by the Danish Cancer Registry had in 5% of cases no histological verification of diagnosis. Furthermore, patients with abdominal tumours, possible ovarian cancer, were registered as ovarian cancer in the Danish Cancer Registry, but not in the DACOVA register. This explains some of the differences between the number of ovarian cancer registered in the DACOVA register and in the Danish Cancer Registry.

Criteria for protocol allocation were:
(1) Below 70 years of age.
(2) Epithelial ovarian cancer with histological verification of diagnosis.
(3) FIGO stages Ib, Ic, IIa, IIb, IIc, III or IV.

Criteria for exclusion were:
(1) Medical contraindication for postoperative treatment.
(2) Concomitant malignant disease during the last 5 years, except basocellular carcinoma of the skin or carcinoma *in situ* of the uterine cervix.
(3) Refusal to participate in randomised trials.

Histological typing was performed according to WHO 'Common Epithelial Tumours' (Scully, 1977) and histological grading according to the percentage of solid tissue (Mauch *et al.*, 1980): well-differentiated less than 10% (grade I); moderately differentiated more than 10% and less than 50% (grade II); poorly differentiated more than 50% (grade III). All randomised patients had a routine pathological review by an experienced gynaecologic pathologist. FIGO classification was performed according to the 1976 classification.

### Surgery

Surgery was performed according to protocol guidelines and consisted of bilateral salpingo-oophorectomy, hysterectomy

Received 2 January 1991; and in revised form 8 May 1991.

and omentectomy whenever possible. For patients with advanced cancer an attempt was always made to do debulking surgery. At operation meticulous staging was made.

*Postoperative treatment*

Patients with FIGO stages Ib, Ic, IIa, IIb or IIc allocated to protocol were randomised to whole abdominal irradiation or pelvic irradiation + cyclophosphamide. Whole abdominal irradiation consisted of 22.50 Gy over 10 fractions to the abdomen + pelvic boost 22.50 Gy over 10 fractions. Patients treated with pelvic irradiation had 45.00 Gy over 20 fractions to the true pelvis. The technique was a modification of the technique used by Dembo and Bush (Dembo *et al.*, 1979). The dose of cyclophosphamide was 200 mg $m^{-2}$ for 5 days every 4 weeks for 12 cycles. One year after randomisation patients without clinical evidence of disease had a second-look laparotomy.

Patients with stage III or IV allocated to protocol were randomised to cyclophosphamide and cisplatinum (CP) or cyclophosphamide, cisplatinum and doxorubicin (CAP). The doses were: cyclophosphamide 500 mg $m^{-2}$; cisplatinum 60 mg $m^{-2}$ and doxorubicin 40 mg $m^{-2}$. Chemotherapy was given every 4 weeks and repeated 12 times. Patients in clinical response had a second-look laparotomy 1 month after the last cycle of chemotherapy.

Both randomised studies showed no survival differences between the regimens (Sell *et al.*, 1990; Bertelsen *et al.*, 1987).

Non randomised patients in early stages were mainly treated with a combination of pelvic irradiation and cyclophosphamide, or with cisplatinum containing chemotherapy, if they had postoperative therapy at all. Advanced stages were treated with different chemotherapy regimens.

*Follow-up*

Follow-up information was collected prospectively for all randomised patients. For non randomised patients information about primary surgery and pathology was collected prospectively, and information about treatment, recurrence, and survival was obtained retrospectively. Cut-off day was July 1990. Observation time is 68–106 months. No patient was lost to follow-up.

*Statistical method*

Survival was estimated by the Kaplan Meier method and tested for differences by the Mantel Haenzel test. Survival was calculated from the date of operation to the date of death or 1 July 1990 – whichever occurred first.

*Exclusion reasons*

The reasons for exclusions were divided in unavoidable and avoidable reasons. Exclusion criteria foreseen in the protocol, postoperative death, diagnosis at autopsy, psychiatric disease, and doubt about the histologic diagnosis at start of postoperative treatment were considered as unavoidable exclusion reasons. Exclusion of patients because of medical condition not allowing chemo-radiotherapy is of course a subjective decision. But the majority of patients excluded by medical contraindication had very advanced disease.

*DATA calculation*

It was estimated that patient accrual for the adjuvant protocol would be 50 patients per year and for the chemotherapy protocol 100 patients per year. The number required for a statistically significant difference of 15% was calculated to 300 patients in both trials.

**Results**

Between September 1981 and November 1984, 716 patients with epithelial ovarian cancer were registered (Table I). Twenty-three per cent was classified as stage I, 11% as stage II, 48% as stage III, and 15% as stage IV. The FIGO distribution showed minor statistically insignificant differences between the various centres. One hundred and four patients were classified as stage Ia, 131 of the remaining patients were older than 70 years. After exclusion of these 235 patients a total of 481 theoretically fulfilled protocol criterial (Table II). Three hundred and thirty-seven or 70% of the 481 eligible patients were included in the randomised trials. The total number of eligible patients and the number of randomised patients varied a little from year to year. The first year 68% was randomised, the second 74%, and the third 70%.

In early stages 120 patients fulfilled inclusion criteria (Table II), but only 60% (72) were randomised. In advanced stages 361 patients fulfilled inclusion criteria, 73% (265) were randomised. The difference between randomisation percentage in early and advanced stages were statistically significant ($\chi^2 = 7.718$, d.f. = 1, $P = 0.0055$).

*Treatment*

Five patients had no laparotomy: four non randomised patients, one diagnosed at laparoscopy and three at post-mortem examination, and one randomised patients with vaginal metastases. Another 14 patients with advanced tumour had no postoperative treatment and died within 1 month after explorative laparotomy. After exclusion of these 19 patients 462 remained for analysis of postoperative treatment. Stages I and II still comprised 120 patients, but stages III and IV now comprised only 342 patients. Patients in stage Ib, Ic, and II allocated to protocol had in 49% whole abdominal irradiation and in 51% pelvic irradiation plus cyclophosphamide (Table III). The preferred treatment for non randomised patients in stage I and II was pelvic irradiation plus cyclophosphamide, which was given in 31%, and cisplatinum containing chemotherapy which was given in 27%; 23% had no postoperative treatment. Advanced staged allocated to protocol had in 51% CAP and in 49% CP. Non randomised patients were in 47% treated with cisplatinum containing poly-chemotherapy. Single drug alkylating agent was given in 22%, 24% had no postoperative treatment at all.

*Survival*

Survival for all patients showed that randomised patients had a better survival than non randomised patients (Figure 1). The difference was statistically significant ($P = 0.0005$). For stages Ib, Ic or II there were no difference between survival in randomised and non randomised patients ($P = 0.45$) but stages III and IV randomised patients had a statistically significant better survival than non randomised patients ($P = 0.0002$). The survival superiority in randomised patients disappeared when groups with similar stages and treatments were compared. In stages III and IV there was no difference in survival when only patients treated with combination chemotherapy were compared (Figure 2) ($P = 0.98$).

*Exclusion reasons*

In stages Ib, Ic, and II 60% were randomised, 11% were excluded because of unavoidable reasons and 29% because of avoidable reasons (Table II). In contrast 73% were randomised in stages III and IV. Twenty-one per cent was excluded because of unavoidable reasons and only 6% because of avoidable reasons. The difference between early stages and advanced stages in the percentage of avoidable excluded patients was statistically significant ($\chi^2 = 35.080$, d.f. = 1, $P = 0.0000$). Medical contraindication and refusal were unavoidable exclusion reasons for seven out of 13 patients in

Table I FIGO classification distributed according to oncologic centre

| FIGO stage | Centre A n | A (%) | B n | B (%) | C n | C (%) | D n | D (%) | Total n | Total (%) |
|---|---|---|---|---|---|---|---|---|---|---|
| Iai | 3 | (4) | 27 | (9) | 21 | (10) | 17 | (12) | 68 | (10) |
| Iaii | 5 | (7) | 13 | (5) | 10 | (5) | 8 | (6) | 36 | (5) |
| Ibi | 2 | (3) | 3 | (1) | 5 | (2) | 0 | (0) | 10 | (1) |
| Ibii | 0 | (0) | 1 | (0) | 7 | (3) | 0 | (0) | 8 | (1) |
| Ic | 6 | (8) | 15 | (5) | 13 | (6) | 12 | (9) | 46 | (6) |
| IIa | 1 | (1) | 7 | (2) | 5 | (2) | 2 | (1) | 15 | (2) |
| IIb | 3 | (4) | 15 | (5) | 10 | (5) | 6 | (4) | 34 | (5) |
| IIc | 2 | (3) | 16 | (6) | 9 | (4) | 2 | (1) | 29 | (4) |
| III | 47 | (65) | 133 | (46) | 90 | (42) | 76 | (55) | 346 | (48) |
| IV | 3 | (4) | 55 | (19) | 37 | (17) | 14 | (10) | 109 | (15) |
| No laparotomy | 0 | (0) | 5 | (2) | 8 | (4) | 2 | (1) | 15 | (2) |
| Total | 72 | | 290 | | 215 | | 139 | | 716 | |

Table II Reasons for exclusions distributed by protocol

| | FIGO stages Ib,Ic,II | III, IV | Total |
|---|---|---|---|
| Randomised | 72 60% | 265 73% | 337 70% |
| Non randomised | 48 40% | 96 27% | 144 30% |
| Reasons for exclusions | | | |
| Unavoidable | 13 11% | 75 21% | 88 18% |
| Other cancer | 0 | 14 | 14 |
| Previous irradiation | 2 | 2 | 4 |
| Medical contraindication | 4 | 39 | 43 |
| Dead postoperatively | 0 | 3 | 3 |
| Diagnosed at autopsy | 0 | 3 | 3 |
| Uncertain histology | 2 | 3 | 5 |
| Psychiatric disease | 2 | 1 | 3 |
| Refusal | 3 | 10 | 13 |
| Avoidable | 35 29% | 21 6% | 56 12% |
| Insufficient staging | 11 | 2 | 13 |
| Wrong FIGO stage | 17 | 0 | 17 |
| Misunderstanding of criteria | 3 | 10 | 13 |
| Chemotherapy preoperatively | 1 | 4 | 5 |
| Delayed trial start | 0 | 2 | 2 |
| Physicians decision | 2 | 1 | 3 |
| Various | 1 | 2 | 3 |

early stages. The most frequent avoidable exclusion reason for early stages was wrong FIGO classification, which occurred among 17 out of 35 cases. Fifteen patients with unilateral tumour and ascites without tumour cells were classified as stage Ia after surgery. The correct classification was Ic. Two cases in stage IIb were primarily classified as stage III and allocated to the protocol for stages III and IV. Eleven patients were excluded because of insufficient staging. In advanced stages 39 out of 75 unavoidable excluded patients, were not randomised because of medical condition not allowing chemotherapy. WHO performance score was in several patients 3 or 4, 11 died within 1 month after diagnosis. The most frequent avoidable exclusion reasons for stages III and IV was misunderstanding of protocol inclusion and exclusion criteria. One centre had, in contradiction to protocol guidelines, the opinion, that patients only explorative laparotomis-

Figure 1 Survival in 481 patients fulfilling randomisation criteria distributed according to protocol.

Figure 2 Survival in 281 stages III and IV patients treated with cisplatinum polychemotherapy, distributed in randomised (264), and non randomised patients (37).

ed should not be randomised. Eight patients were excluded because of this reason.

The percentage of randomised patients showed a statisti-

Table III Distribution of treatment in relation to protocol and randomisation

| Treatment | Ib, Ic and II Randomised Yes n | (%) | No n | (%) | III and IV Randomised Yes n | (%) | No n | (%) |
|---|---|---|---|---|---|---|---|---|
| Whole Abd.RT | 35 | (49) | 1 | (2) | 0 | (0) | 0 | (0) |
| Pelvic RT | 0 | (0) | 2 | (4) | 0 | (0) | 1 | (1) |
| Pelvic RT + Cyclo | 37 | (51) | 15 | (31) | 0 | (0) | 4 | (5) |
| Alkylating single drug | 0 | (0) | 6 | (13) | 0 | (0) | 17 | (22) |
| Cisplatin polychemotherapy | 0 | (0) | 13 | (27) | 264 | (100) | 37 | (47) |
| No treatment | 0 | (0) | 11 | (23) | 0 | (0) | 19 | (24) |
| | 72 | | 48 | | 264* | | 78 | |

*19 patients excluded, five without laparotomy and 14 died within a month after exploratory laparotomy.

cally insignificant difference between centres A and the remaining centres. Centre A included only 43% in the protocol for early stages and 58% in the protocol for advanced stages *vs* 62% and 75% for the remaining centres (Table IV) (*P* values 0.38 and 0.10 respectively). However, at centre A statistically significant more patients were excluded because of avoidable exclusion reasons than at the other centres, 31% *vs* 9% (Table V) ($\chi^2 = 23.274$, d.f. = 1, $P = 0.0000$).

The relationship between randomisation and characteristics of patients eligible for the early stage protocol is shown in Table VI. Patients in FIGO stage Ic, with mucinous carcinoma, residual tumour larger than 1 cm in diameter, or ascites had a lower frequency of randomisation than the total group. Stage Ic was, as mentioned above, not randomised because of misinterpretation of the FIGO classification. The central DACOVA register changed the classification from Ia to Ic for 15 patients with ascites exceeding 100 ml. Patients with mucinous carcinoma or with residual tumour larger than 1 cm in diameter or ascites were excluded because the physician in charge thought that the protocol treatment would be inappropriate. Only 35% of the eligible patients with residual tumour larger than 1 cm was randomised. The relationship between randomisation and characteristics of patients eligible for the advanced stage protocol is shown in Table VII. FIGO stage, histological type, size of residual tumour and the presence of ascites had no influence on protocol allocation.

**Table IV** Protocol allocation, distribution by centre and protocol

| | *FIGO stages* | | | | | |
|---|---|---|---|---|---|---|
| | *Ib, Ic and II* | | | *III and IV* | | |
| | *Randomised* | | | *Randomised* | | |
| | *Yes* | | *No* | *Yes* | | *No* |
| | *n* | *(%)* | *n* | *n* | *(%)* | *n* |
| Centres | | | | | | |
| A | 6 | (43) | 8 | 23 | (58)· | 17 |
| B | 26 | (56) | 20 | 115 | (77) | 34 |
| C | 27 | (68) | 13 | 76 | (75) | 26 |
| D | 13 | (65) | 7 | 51 | (73) | 19 |
| Total | 72 | | 48 | 265 | | 96 |

$\chi^2 = 3.092$, d.f. = 3, $P = 0.38$; $\chi^2 = 6.346$, d.f. = 3, $P = 0.10$.

**Table V** Protocol allocation distributed by centre and by unavoidable/avoidable exclusion reasons

| | *Randomised* | | | | *Non randomised* | | | |
|---|---|---|---|---|---|---|---|---|
| | *Yes* | | *No* | | *Unavoidably* | | *Avoidably* | |
| | *n* | *(%)* | *n* | *(%)* | *n* | *(%)* | *n* | *(%)* |
| A | 29 | (54) | 25 | (46) | 8 | (15) | 17 | (31) |
| B | 141 | (72) | 54 | (28) | 37 | (20) | 17 | (8) |
| C | 103 | (73) | 39 | (27) | 25 | (17) | 14 | (10) |
| D | 64 | (71) | 26 | (29) | 18 | (20) | 8 | (9) |
| | 337 | | 144 | | 88 | | 56 | |

**Table VI** Randomisation in relation to charactistics of 120 FIGO stage Ib, Ic, and II patients

| | *Randomised* | | |
|---|---|---|---|
| | *Yes* | | *No* |
| | *n* | *(%)* | *n* |
| Number of patients | 72 | (60) | 48 |
| FIGO stage | | | |
| Ib | 13 | (87) | 2 |
| Ic | 19 | (48) | 21 |
| IIa | 10 | (71) | 4 |
| IIb | 15 | (58) | 11 |
| IIc | 15 | (60) | 10 |
| Histological type | | | |
| Serous carcinoma | 29 | (69) | 13 |
| Mucinous carcinoma | 6 | (38) | 10 |
| Endometrioid carcinoma | 26 | (63) | 15 |
| Clear cell carcinoma | 4 | (67) | 2 |
| Undifferentiated carcinoma | 6 | (67) | 3 |
| Mixed carcinoma | 1 | (17) | 5 |
| Residual tumour (Stage IIb and IIc) | | | |
| None | 17 | (77) | 5 |
| ⩽1 cm | 7 | (70) | 3 |
| >1 cm | 6 | (35) | 11 |
| Unknown | 0 | (0) | 2 |
| Ascites | 15 | (37) | 26 |
| No ascites | 57 | (72) | 22 |
| Tumour cells in ascites/washings | 20 | (77) | 6 |
| No tumour cells | 23 | (49) | 24 |
| Not examined | 29 | (62) | 18 |

### Discussion

Four hundred and eighty-one patients were theoretically eligible for randomisation in these multicentre trials for epithelial ovarian cancer, but only 70% were randomised. The stage distribution and thereby the number of patients available for randomisation depends upon staging procedure. Careful staging reduces the number of patients in early stages. One centre had in fact a lower frequency of stage Iai. The main reason for this is probably incomplete registration of non randomised patients and not an inferior staging procedure. The study showed statistically significant differences in acceptance and exclusion reasons of the two protocols. For early stages, Ib, Ic or II, only 60% were randomised, 11% were excluded because of unavoidable reasons and as much as 29% because of avoidable reasons. Twenty-eight among 48 excluded patients were not randomised because of wrong FIGO classification or insufficient staging. In stages III or IV 73% of the eligible patients were randomised, 21% were excluded because of unavoidable and only 6% because of avoidable reasons. Medical condition not allowing chemotherapy was the most frequent unavoidable exclusion reason. Eighty-nine per cent of early stages and 79% of advanced stages could have been randomised if all patients excluded by avoidable reasons, had been included. Allocation to the early stages trial was not satisfactory. The number of randomised patients included only two thirds of the available patients. For advanced stages almost all available patients were randomised. The difference is probably explained by a difference in the trial question and not by a difference in administration and monitoring of the trials. The chemotherapy protocol was a simple question and all the investigators felt a need for new treatment and better results. The question of protocol for the early stages was more complicated. The treatment was rather toxic, considered adjuvant, and many investigators doubted the efficacy of whole abdominal irradiation.

**Table VII** Randomisation in relation to characteristics of 342* FIGO stage III and IV patients

| | *Randomised* | | |
|---|---|---|---|
| | *Yes* | | *No* |
| | *n* | *(%)* | *n* |
| Number of patients | 264* | (77) | 78 |
| FIGO stage | | | |
| III | 207 | (78) | 59 |
| IV | 57 | (75) | 19 |
| Histologic type | | | |
| Serous carcinoma | 154 | (80) | 38 |
| Mucinous carcinoma | 18 | (64) | 10 |
| Endometrioid carcinoma | 43 | (77) | 13 |
| Clear cell carcinoma | 7 | (88) | 1 |
| Undifferentiated carcinoma | 35 | (71) | 14 |
| Mixed carcinoma | 7 | (78) | 2 |
| Residual tumour | | | |
| None | 24 | (75) | 8 |
| ⩽1 cm | 43 | (88) | 6 |
| >1 cm | 187 | (75) | 61 |
| Unknown | 10 | (77) | 3 |
| Ascites | 183 | (77) | 56 |
| No ascites | 81 | (79) | 22 |
| Tumour cell in ascites/washings | 147 | (81) | 34 |
| No tumour cells | 54 | (68) | 25 |
| Not examined | 63 | (77) | 19 |

*19 excluded, five without laparotomy and 14 dead within a month after laparotomy.

Furthermore the present study suggested that allocation to the early stage protocol varied with centre, FIGO stage, histological type, and residual tumour. Stage Ic was excluded because of a general misclassification of patients with ascites without tumour cells. The remaining patients were excluded for various subjective reasons. The randomised population was then a selected subset of the total patient population. It may be that the results are true only in this subset and for not all stages Ib, Ic and II. Allocation to the protocol for advanced stages varied with centre, but not with other factors; the main exclusion reason was medical condition not allowing chemotherapy. Centre A excluded a statistically significant greater number of patients because of avoidable reasons than the other centres.

The percentage of randomised patients in the present study was similar to the percentage of eligible patients randomised in other Danish multicentre trials. The Danish colo-rectal multicentre study group included 57% of eligible patients in a randomised study testing the efficacy of postoperative irradiation in colo-rectal cancer Dukes stage B and C (Kronborg *et al.*, 1988). The majority (92%), was excluded because of reasons agreed upon beforehand. However, this trial had a very long list of protocol exclusion criteria. In the Danish Breast Cancer Cooperative Group 20–30% of patients below 70 years of age were not included in the various protocols (West Andersen *et al.*, 1988).

Survival of randomised patients was better than survival of non randomised patients. This is probably caused by selection of patients with better prognosis for randomised studies. Non randomised patients were patients with bad prognosis. The difference in survival disappeared when groups with similar prognostic characteristics and similar treatments were compared. Thus, advanced stages treated with cisplatinum containing chemotherapy showed no survival difference between randomised and non randomised patients.

For early stages the number of randomised patients was below the estimated number, 20 *vs* 50 per year. For advanced stages the estimate was correct. Both trials were unable to show a statistically significant survival difference between the randomisation groups. The protocol for early stages stopped in November 1988. A study testing the effect of adjuvant treatment in early ovarian cancer will require participation of a large number or departments because of the small number of patients in stages I and II. The chemotherapy protocol stopped in November 1984 after inclusion of 265 patients. Recently this trial has been analysed together with four other trials randomising between CAP and CP (Ovarian Cancer Meta- Analysis Group, 1991). The five trials included 1,194 patients. A statistically significant difference for 5 year survival in favour of the CAP regimen was observed ($P = 0.02$). The observed difference was 6% and not 15% as estimated. Considering the poor survival of advanced ovarian cancer the 6% improvement is of clinical interest. A realistic estimate of the expected difference at the beginning of the trial would have demonstrated that a large scale study with participation of departments outside Denmark was necessary. A large scale trial had required a different study design recording only very few data.

The present study showed that the patient allocation to a multicentre trial is influenced by many factors. In setting up randomised trials the following should be considered.

(1) Only essential and simple questions should be examined in multicentre trials.
(2) Realistic data estimates should be performed. Patient accrual and the difference between treatments groups are usually overestimated. A statistically significant difference will very often require inclusion of a large number of patients.
(3) Participation of departments randomising a small and selected part of their patients is questionable. Results based on data from these selected patients may be of no use in clinical pratice.

We are grateful to the DACOVA group and the Danish Cancer Society which supported the study with grants.

### References

BERTELSEN, K., JAKOBSEN, A., ANDERSEN, J.E. & 21 others (1987). A randomised study of cyclophosphamide and cisplatinum with or without doxorubicin in advanced ovarian carcinoma. *Gynecol. Oncol.*, **28**, 161.

DANISH CANCER REGISTRY (1984). *Cancer Incidence in Denmark*, 16 & 42.

DEMBO, A.J., VAN DYK, J., JAPP, B. & 4 others (1979). Whole abdominal irradiation by a moving-strip technique for patients with ovarian cancer. *Int. J. Radiat. Oncol. Biol. Phys.*, **5**, 1933.

KRONBORG, O., FENGER, C., BERTELSEN, K. & 11 others (1988). Escape clauses in a multicentre trial – unforeseen problems and possible influence upon general validity of conclusions. *Theor. Surg.*, **2**, 157.

MAUCH, P.M., EHRMANN, R.L., GRIFFITHS, C.T., MARCK, A., KNAPP, R.C. & LEVENES, M.B. (1980). Radiation therapy in stage II ovarian carcinoma. *Cancer*, **45**, 1344.

OVARIAN CANCER META-ANALYSIS GROUP CP versus CAP chemotherapy of ovarian carcinoma: a meta-analysis. Accepted, *J. Clin. Oncol.*, March 1991.

SCULLY, R.E. (1977). Ovarian tumors: a review. *Am. J. Pathol.*, **87**, 686.

SELL, A., BERTELSEN, K., ANDERSEN, J.E., STRØYER, I. & PANDURO, J. (1990). Randomized study of whole abdominal irradiation versus pelvic irradiation plus cyclophosphamide in treatment of early ovarian cancer. *Gynecol. Oncol.*, **37**, 367.

WEST ANDERSEN, K. & MOURIDSEN, H.T. (1988). Danish Breast Cancer Cooperative Group (DBCG). A description of the nationwide programme for primary breast cancer. *Acta. Oncol.*, **27**, 627.